# Faulty homocysteine recycling in diabetic retinopathy

**DOI:** 10.1186/s40662-019-0167-9

**Published:** 2020-01-10

**Authors:** Renu A. Kowluru, Ghulam Mohammad, Nikhil Sahajpal

**Affiliations:** Department of Ophthalmology, Visual Sciences and Anatomical Sciences, Wayne State University, 4717 St. Antoine, Detroit, MI 48201 USA

**Keywords:** Diabetic retinopathy, DNA methylation, Homocysteine, Hydrogen sulfide, Mitophagy, Epigenetics, Mitochondria, Oxidative stress, Retina

## Abstract

**Background:**

Although hyperglycemia is the main instigator in the development of diabetic retinopathy, elevated circulating levels of a non-protein amino acid, homocysteine, are also associated with an increased risk of retinopathy. Homocysteine is recycled back to methionine by methylenetetrahydrofolate reductase (MTHFR) and/or transsulfurated by cystathionine β-synthase (CBS) to form cysteine. CBS and other transsulfuration enzyme cystathionine-γ-lyase (CSE), through desulfuration, generates H_2_S. Methionine cycle also regulates DNA methylation, an epigenetic modification associated with the gene suppression. The aim of this study was to investigate homocysteine and its metabolism in diabetic retinopathy.

**Methods:**

Homocysteine and H_2_S levels were analyzed in the retina, and CBS, CSE and MTHFR in the retinal microvasculature from human donors with established diabetic retinopathy. Mitochondrial damage was evaluated in retinal microvessels by quantifying enzymes responsible for maintaining mitochondrial dynamics (fission-fusion-mitophagy). DNA methylation status of *CBS* and *MTHFR* promoters was examined using methylated DNA immunoprecipitation technique. The direct effect of homocysteine on mitochondrial damage was confirmed in human retinal endothelial cells (HRECs) incubated with 100 μM L-homocysteine.

**Results:**

Compared to age-matched nondiabetic control human donors, retina from donors with established diabetic retinopathy had ~ 3-fold higher homocysteine levels and ~ 50% lower H_2_S levels. The enzymes important for both transsulfuration and remethylation of homocysteine including CBS, CSE and MTHFR, were 40–60% lower in the retinal microvasculature from diabetic retinopathy donors. While the mitochondrial fission protein, dynamin related protein 1, and mitophagy markers optineurin and microtubule-associated protein 1A/1B-light chain 3 (LC3), were upregulated, the fusion protein mitofusin 2 was downregulated. In the same retinal microvessel preparations from donors with diabetic retinopathy, DNA at the promoters of *CBS* and *MTHFR* were hypermethylated. Incubation of HRECs with homocysteine increased reactive oxygen species and decreased transcripts of mtDNA-encoded *CYTB*.

**Conclusions:**

Compromised transsulfuration and remethylation processes play an important role in the poor removal of retinal homocysteine in diabetic patients. Thus, regulation of their homocysteine levels should ameliorate retinal mitochondrial damage, and by regulating DNA methylation status of the enzymes responsible for homocysteine transsulfuration and remethylation, should prevent excess accumulation of homocysteine.

## Introduction

Diabetic retinopathy remains the leading cause of vision loss in working age adults. Many molecular mechanisms have been implicated in its development, but despite ongoing cutting edge research in the field, the molecular mechanism of this multi-factorial disease is still not clear [1]. In the pathogenesis of diabetic retinopathy, oxidative stress is increased in the retina and its vasculature, mitochondria are damaged and have impaired homeostasis, gene transcription associated with oxidative stress are altered, and apoptosis of capillary cells are accelerated [2–5].

Experimental and clinical studies have documented that diabetic patients and animal models have elevated circulating levels of homocysteine, a sulfur-containing amino acid [6]. High plasma homocysteine levels are associated with endothelial dysfunction, and in diabetic patients, with many complications including nephropathy, cardiomyopathy and neuropathy [7–9]. Studies using genetically manipulated mice that can accumulate homocysteine have suggested a role for homocysteine in diabetic retinopathy; these animals have impaired visual function and damaged blood retinal barrier [10, 11]. Homocysteine was also shown to induce mitochondrial dysfunction, and in retinal ganglion cells, it was implicated in the dysregulation of mitochondrial dynamics [12]. Experimental models of diabetic retinopathy have clearly documented the role of mitochondrial homeostasis in the development of diabetic retinopathy; retinal mitochondria were damaged in diabetes, their copy numbers were decreased, and while the mitochondrial fusion marker, mitofusin 2 (Mfn2), was downregulated, mitophagy markers were upregulated, and capillary cell apoptosis was accelerated [3, 4, 13, 14].

Homocysteine is a non-protein amino acid and is biosynthesized from methionine by S-adenosyl-methionine synthetase, forming *S*-adenosyl methionine (SAM). Homocysteine formed can either be remethylated back to L-methionine, or via transsulfuration, to L-cysteine, and cysteine is an important amino acid for the biosynthesis of glutathione (GSH). Enzymatically, methylenetetrahydrofolate reductase (MTHFR) converts homocysteine to methionine, and CBS catalyzes the condensation of homocysteine with serine to form cystathionine, which can be further converted to L-cysteine [15–17]. In addition to cysteine being a substrate for the biosynthesis of glutathione, it also serves as a substrate for CBS and cystathionine-*γ* lyase (CSE) to produce hydrogen sulfide (H_2_S) via a desulfuration reaction [16]. H_2_S is now considered as the third gasotransmitter with important roles in reducing oxidative stress and inflammation, and also regulating apoptosis [18]. In the pathogenesis of diabetic retinopathy, retinal oxidative stress and inflammation are increased and GSH levels are decreased [3, 4, 19–21]. However, what happens to homocysteine, and its metabolizing machinery in the retina of diabetic retinopathy patients is not clear.

The aim of this study was to investigate homocysteine and its metabolism in diabetic retinopathy. Homocysteine and the machinery essential for its removal, and mitochondrial damage was investigated in the retina and its vasculature from human donors with established diabetic retinopathy. The effect of homocysteine on oxidative stress and mitochondrial damage was confirmed in human retinal endothelial cells (HRECs) incubated in the presence of supplemental homocysteine.

## Methods

### Human donor

Human postmortem eyes globes, enucleated within 6–8 h of death, from donors with clinically documented diabetic retinopathy, were supplied on ice by the Eversight Eye Bank, Ann Arbor, MI, USA. The retina was isolated and immediately used for microvessel preparation. These donors ranged from 55 to 75 years of age, and the duration of diabetes was from 10 to 41 years (Table 1). Age- and sex-matched nondiabetic donors were used as controls. The diabetic retinopathy group had nine donors, and nondiabetic group had eight donors. The eye globes were coded by the Eye Bank and did not contain any patient identification; this met the criteria for ‘exemption’ from Wayne State University’s Institutional Review Board.

**Table 1:** Age and duration of diabetes of human donors

	Age (years)	Duration of diabetes (years)
Nondiabetic donors		
1	68	–
2	52	–
3	71	–
4	72	–
5	63	–
6	74	–
7	65	–
8	75	–
Donors with diabetic retinopathy		
1	55	35
2	70	> 20
3	71	41
4	75	35
5	73	25
6	75	25
7	68	16
8	61	10
9	61	22

A small portion (1/6th to 1/4th) of the whole retina was subjected to osmotic shock by incubating it in 10–15 ml of distilled water for 1 h at 37 °C with gentle shaking. Microvessels were then isolated from the retina by repeated inspiration and ejection using Pasteur pipette under a microscope, and were then rinsed with sterile PBS [22–24]. As reported previously [25], these microvessel preparations are largely devoid of any nonvascular components. However, due to the exposure of the retina to hypotonic shock, cytosolic components are lost.

### Retinal endothelial cells

Human retinal endothelial cells (HRECs) were purchased from Cell Systems Corporation (Cat. No. ACBRI 181, Cell Systems Corp, Kirkland, WA, USA), and were cultured in Dulbecco’s modified Eagle medium (DMEM)-F12 containing 12% heat-inactivated fetal bovine serum and 15 μg/ml endothelial cell growth supplement_,_ as described previously [26, 27]. Cells from the 7th–8th passage were incubated in the DMEM incubation medium containing reduced serum and growth supplement (2% and 2 μg/ml, respectively) for 48 h in the presence or absence of 100 μM L-Homocysteine thiolactone hydrochloride (Cat No**. S784036**, Sigma-Aldrich, St Louis, MO) [10], and were analyzed for mitochondrial damage. Incubation of HRECs with homocysteine for 48 h had no effect on their cell phenotype.

### Gene transcripts

Total RNA was isolated from retinal microvessels or HRECs using TRIzol reagent (Invitrogen, Carlsbad, CA). cDNA was synthesized using a High Capacity cDNA Reverse Transcription kit (Applied Biosystems, Foster City, CA). Quantitative real-time PCR (q-RTPCR) was performed using gene-specific primers (Table 2) by SYBR Green assay in ABI 7500 Cycler detection system (Applied Biosystems), and the specific products were confirmed by SYBR green single melt curve analysis. The results were normalized to the expression of the housekeeping gene β-actin and the relative fold change was calculated using delta Ct method [26, 27].

**Table 2:** Primer sequence

Gene	Sequence (5′-3′)
*CBS*	TCCCCACATCACCACACTGC ATCATCCGCAGGCTGATGCG
*MTHFR*	GAAGTACGAGCTCCGGGTTA AAGATGCCCCAAGTGACAG
*CSE*	AGGTTTCCTGCCACACTTCC TATTCAAAACCCGAGTGCTGG
*CYTB*	TCACCAGACGCCTCAACCGC GCCTCGCCCGATGTGTAGGA
*DRP1*	GAAGGAGGCGAACTGTGGGC GCAGCTGGATGATGTCGGCG
*MFN2*	ATGCAGACGGAAAAGCACTT ACAACGCTCCATGTGCTGCC
*LC3*	TGGTCAAGATCATCCGGCGC GAAGCCGAAGGTTTCCTGGG
*OPTN*	GAGAAGGCTCTGGCTTCCAA GTCATGGTTTCCAGGTCCTCTT
*DNMT1*	AGTCCGATGGAGAGGCTAAG TCCTGAGGTTTCCGTTTGGC
*β-ACTIN*	AGCCTCGCCTTTGCCGATCCG TCTCTTGCTCTGGGCCTCGTCG
*CBS* promoter (− 116 to + 64)	GTGCTCTGCCACGAGACATTGTCACCTGGACGGATACATGGAAA
*MTHFR* promoter (− 406 to − 233)	CCAGCATCAAGTTCTAACCCACAA ATCACCCTCCAGAGAAGGAACAG

### Homocysteine

Levels of homocysteine were measured in the retinal homogenate (15 μg protein) using an ELISA kit from Cell Bio Labs Inc. (Cat No. STA-670, San Diego, CA, USA), according to the protocol provided with the kit. Final absorbance was measured at 450 nm using an ELISA plate reader [28].

### Western blotting

Retinal microvessels (40–50 μg protein) were separated on a 4–20% SDS- polyacrylamide gradient gel (BioRad, Hercules, CA), and transferred to a nitrocellulose membrane. After blocking with 5% non-fat milk for 1 h, the membrane was incubated with the antibodies against the proteins of interest, and β-actin was employed as a loading control (Table 3).

**Table 3:** Antibodies used for protein expression

Protein	Dilution	Cat No.	Source
Homocysteine	1:1000	ab15154	Abcam, Cambridge, MA
CBS	1:1000	PA5–72506	Invitrogen, Carlsbad, CA
DRP1	1:1000	ab184247	Abcam, Cambridge, MA
MFN2	1:1000	ab56889	Abcam, Cambridge, MA
LC3	1:1000	ab58610	Abcam, Cambridge, MA
β- ACTIN	1:2000	ab8227	Abcam, Cambridge, MA

*CBS*= Cystathionine β-synthase; *DRP1*= Dynamin related protein 1; *MFN2*= Mitofusin-2; *LC3*= Microtubule-associated protein 1A/1B-light chain 3

### Cystathionine β synthase activity

CBS activity was measured in the retinal homogenate (50 μg protein) using cystathionine β synthase activity assay kit (Cat No. K998 Bio Vision, Milpitas, CA, USA), following the manufacturer’s protocol. The fluorescence was measured immediately for 60 min at 368 nm excitation and 460 nm emission wavelengths. The specificity of CBS activity was evaluated by performing the assay in the presence of no enzyme, and positive controls.

### Glutathione levels

GSH levels were quantified by an enzymatic recycling method using a GSH Assay Kit (Cat No. 703002; Cayman Chemical, Ann Arbor, MI). Retinal homogenate (7–10 μg protein) was deproteinized by phosphoric acid, and GSH was measured in the supernatant after neutralizing its pH with triethanolamine. The assay is based on the reaction of sulfhydryl group of GSH with 5,5′-dithio-*bis*-2-nitrobenzoic acid, producing 5-thio-2-nitrobenzoic acid, which is measured at 412 nm [19, 29].

### Quantification of methylated cytosine

Genomic DNA was isolated from retinal microvessels using Qiagen DNA isolation kit (Qiagen, Valencia, CA, USA), and was immunoprecipitated with antibodies against 5mC. The levels of 5mC were quantified using methylated DNA Immunoprecipitation (MeDIP) kit (Cat. No. P-1015, EPIGENTEK, Farmingdale, NY, USA) [30]. The enrichment of 5mC at the promoters of *CBS* and *MTHFR* was quantified by q-RTPCR using their gene specific primers.

### Hydrogen sulfide

H_2_S was measured in the retinal homogenate using methods described by others [31]. Briefly, to trap the H_2_S, 50 μg of retinal homogenate in 200 μl PBS was transferred directly into a tube containing 1% zinc acetate and 12% NaOH. Following incubation for 20 min at room temperature, N-dimethyl-p-phenylenediamine sulfate in 7.2 M HCl and FeCl_3_ were added. The mixture was incubated for 15 min at room temperature in the dark and was transferred to a tube containing 10% trichloroacetic acid to precipitate protein. The precipitated protein was removed by centrifugation at 10,000 g for 5 min and the absorbance of the resulting supernatant was measured at 670 nm [31]. H_2_S concentration in each sample was quantified using NaHS as a standard.

### Reactive oxygen species

Total reactive oxygen species (ROS) levels were quantified in HRECs (5 μg protein) using 2′,7′-dichlorofluorescein diacetate (DCFH-DA; Cat. No. D6883; Sigma-Aldrich Corp.), as described previously [26].

### Statistical analysis

Statistical analysis was carried out using Sigma Stat software (Systat Software, Inc. San Jose, CA). Data are presented as mean ± SD of 3 or more experiments, each performed in duplicate. Comparison between groups were made using one-way ANOVA followed by Dunn’s t-test and a *p* value less than 0.05 was considered statistically significant.

## Results

**Fig. 1:**
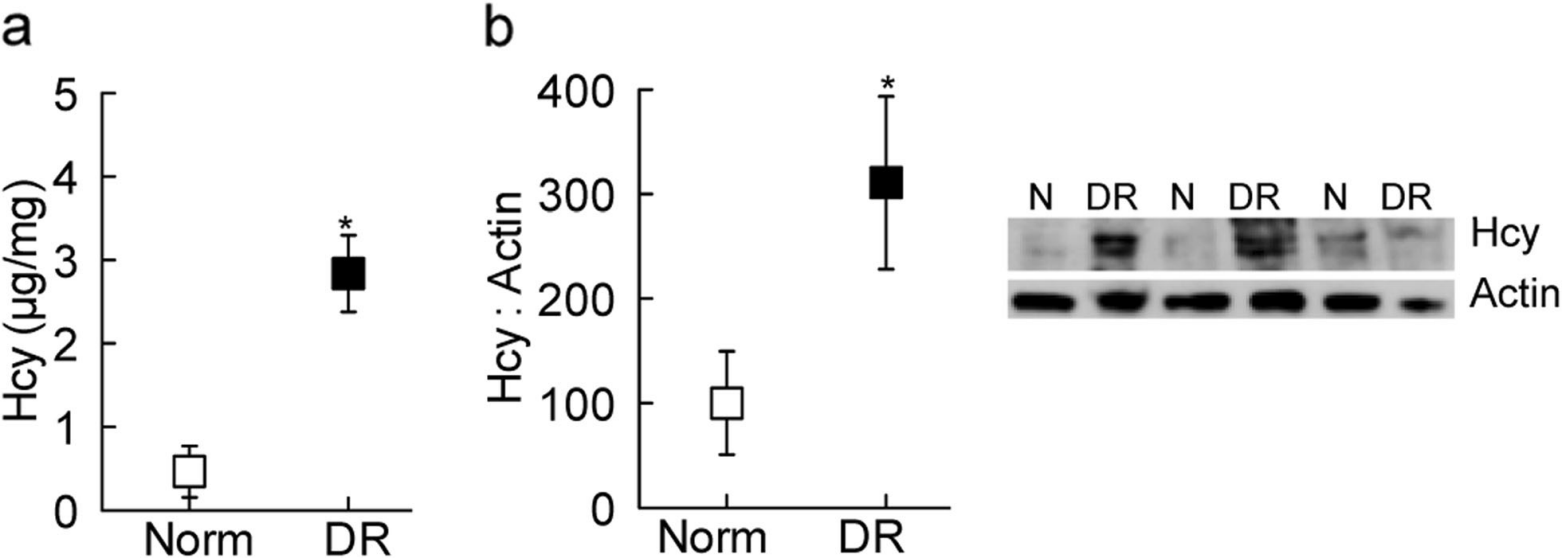
Homocysteine levels in human donors. Homocysteine was measured (**a**) in the retina by an ELISA method, and (**b**) in retinal microvessels by Western blotting using β-actin as a loading protein. Measurements were performed in duplicates in the retina from 6 to 8 human donors with established diabetic retinopathy (DR) and nondiabetic controls (Norm) groups. Data are represented as mean ± SD. *p < 0.05 compared with nondiabetic donors

Homocysteine levels were about three-fold higher in donors with established diabetic retinopathy compared to their age-matched nondiabetic donors (Fig. 1a). A similar increase in homocysteine expression was observed in the retina from diabetic donors with retinopathy by Western blot (Fig. 1b).

**Fig. 2:**
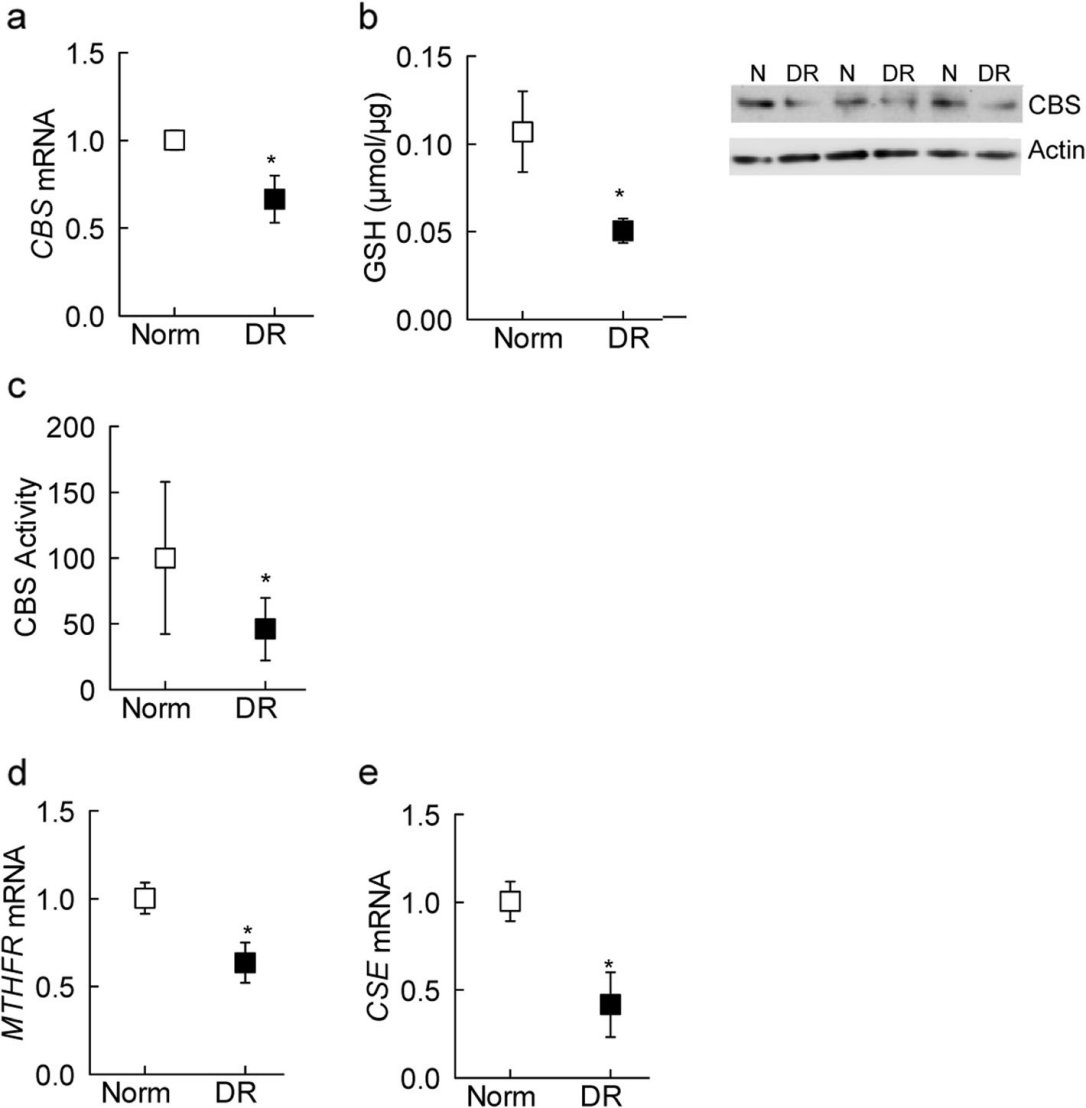
Homocysteine metabolizing machinery in diabetic retinopathy. Retinal microvessels were employed to determine CBS (**a**) gene transcripts by q-RTPCR, (**b**) protein expression by Western blotting, using β-actin as a housekeeping gene and loading protein, respectively, and (**c**) enzyme activity by measuring fluorescence at 368 nm excitation and 460 nm emission wavelengths. Values obtained from nondiabetic controls are considered as 100%. Gene transcripts of (**d**) *MTHFR* and (**e**) *CSE* were quantified by q-RTPCR using β-actin as a housekeeping gene. Data are represented as mean ± SD, obtained from retinal microvessels from 6 to 8 nondiabetic and 7–8 diabetic retinopathy donors. *p < 0.05 vs. nondiabetic donors

Homocysteine can be converted to cystathione by CBS and CSE [16, 17]; CBS and CSE enzymes were determined in the microvessels. Compared to non-diabetic control donors, diabetic retinopathy donors had 40–60% reduction in the gene and protein expressions of CBS, and 60% decrease in CBS enzyme activity (Fig. 2a-c). Consistent with CBS, in the same diabetic retinopathy donors, gene transcripts of *MTHFR* and *CSE* also decreased by 40 and 60%, respectively (Fig. 2d and e).

**Fig. 3:**
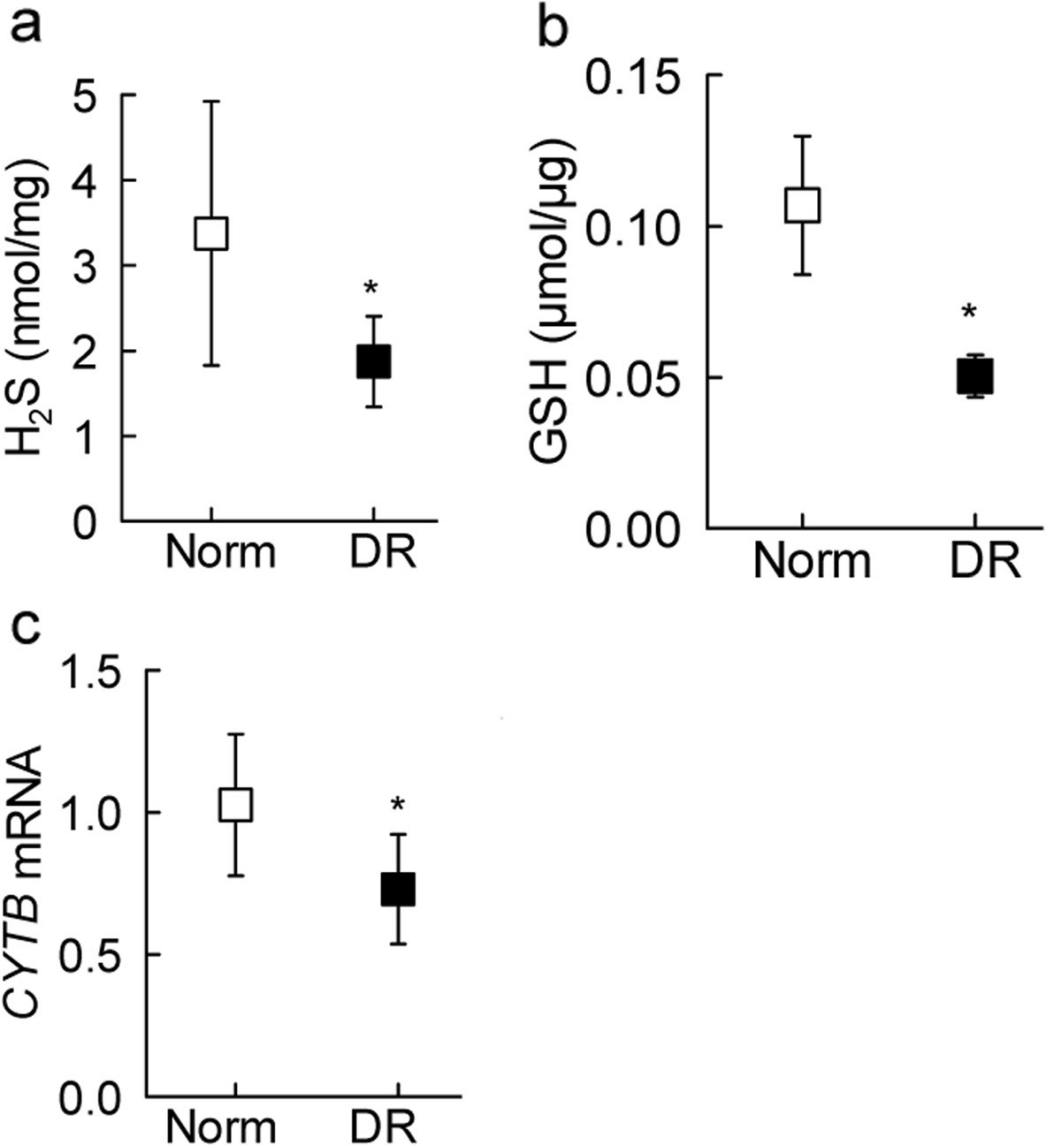
Retinal hydrogen sulfide levels and oxidative stress markers in diabetic retinopathy. Retinal homogenate was used to measure (**a**) H_2_S levels spectrophotometrically at 670 nm using N-dimethyl-p-phenylenediamine sulfate, and (**b**) GSH levels by an enzymatic recycling method. (**c**) Gene transcripts of *CYTB* were quantified in retinal microvessels by q-RTPCR to estimate mtDNA damage. Each measurement was made in duplicates in 5–7 samples each in nondiabetic control (Norm) and diabetic retinopathy (DR) groups. The values obtained from nondiabetic controls are considered as one. *p < 0.05 compared with nondiabetic donors

Since CBS and CSE are also intimately involved in regulating H_2_S levels [16], as with the transsulfuration and remethylation machinery, diabetic retinopathy donors had over a 2-fold decrease in retinal H_2_S levels (Fig. 3a).

Imbalance between homocysteine and H_2_S decreases intracellular antioxidants GSH [32]; Fig. 3b shows ~ 50% decrease in GSH content in diabetic retinopathy donors, compared to their nondiabetic controls. Decrease in GSH shifts the equilibrium of free radicals towards increased oxidative stress and increased free radicals damage mitochondria; consistent with decrease in GSH, mtDNA damage was also significantly higher, as seen by ~ 30% decrease in the gene transcripts of *CYTB* in the retinal microvessels from donors with diabetic retinopathy (Fig. 3c).

**Fig. 4:**
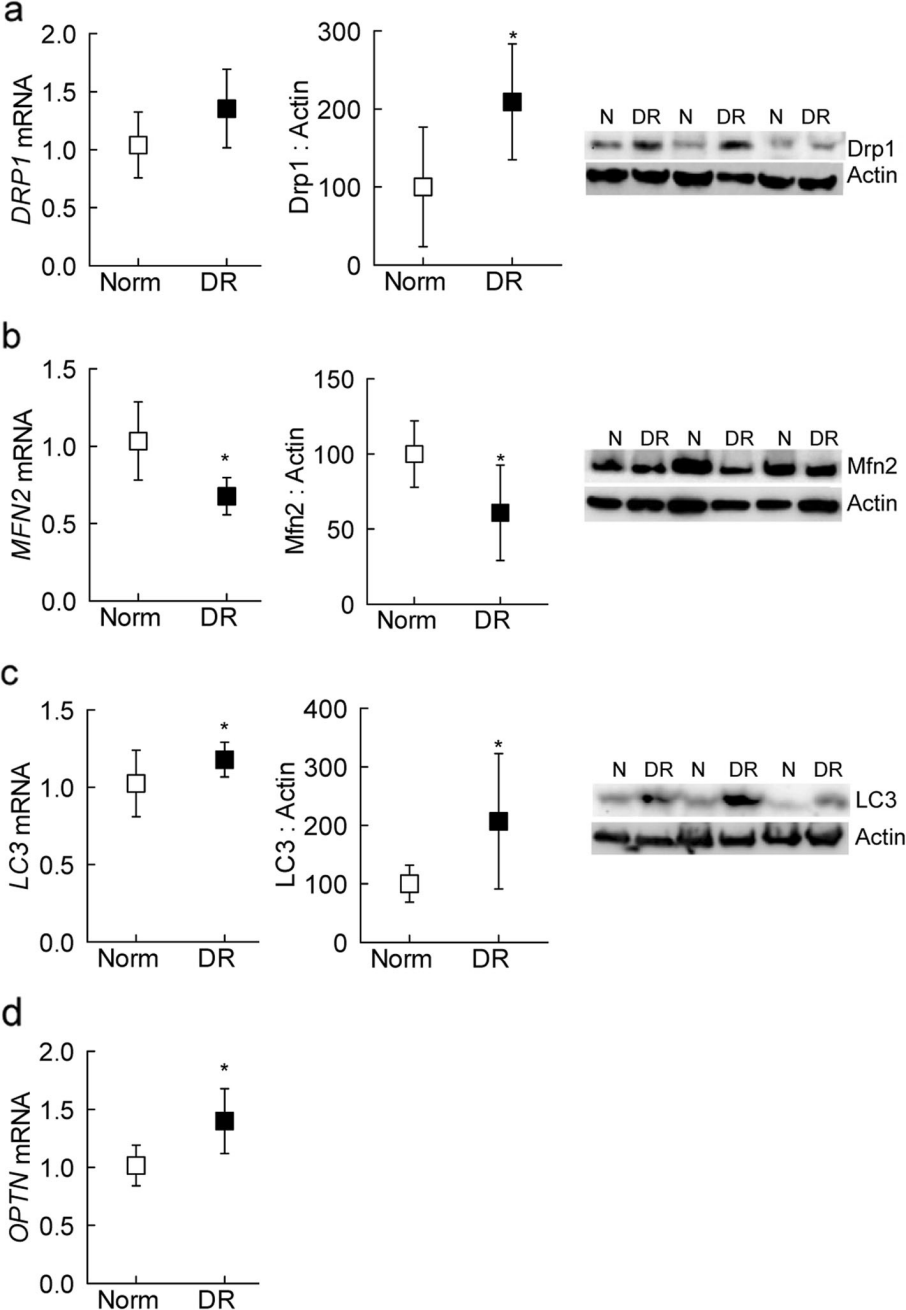
Mitochondrial dynamics in diabetic retinopathy. Retinal microvessels from 6 to 8 donors each with diabetic retinopathy, and nondiabetic controls were analyzed for gene and protein expressions of (**a**) DRP1, (**b**) MFN2, (**c**) LC3 and (**d**) OPTN by q-RTPCR and Western blotting, respectively, using β-actin as a housekeeping gene/loading protein. Gene transcripts and protein expression values obtained from nondiabetic controls are considered as 1 and 100%, respectively

Mitochondrial homeostasis is critical for its proper functioning, and is maintained by fusion-fission-mitophagy [33]. Compared to nondiabetic donors, while gene and protein expressions of DRP1 were increased by ~ 70% in the retinal microvessels from donors with diabetic retinopathy, MFN2 gene and protein expressions decreased by ~ 40% (Fig. 4a and b). Alterations in mitochondrial fusion-fission enzymes were accompanied by increased mitophagy markers including microtubule-associated protein 1A/1B-light chain 3 (LC3) and optineurin (OPTN) in the same retinal microvessel preparations (Fig. 4c and d).

**Fig. 5:**
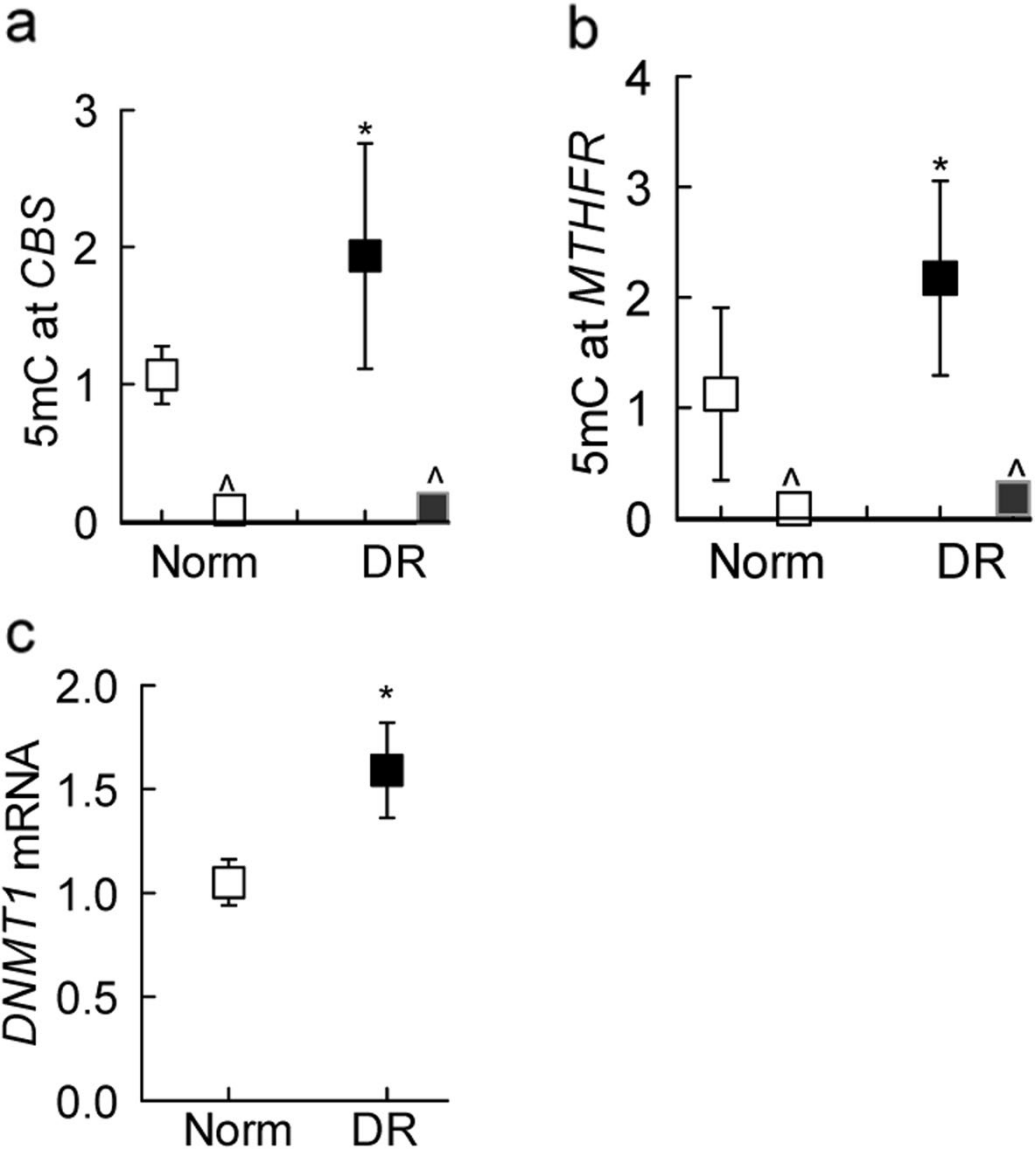
DNA methylation of homocysteine metabolizing enzymes. Isolated genomic DNA from retinal microvessels were utilized to quantify 5mC levels at the promoters of (**a**) *CBS* and (**b**) *MTHFR* using methylated DNA immunoprecipitation and IgG as an antibody control (^). **c** Dnmt1 gene transcripts were measured by q-RTPCR using β-actin as a housekeeping gene. Each measurement was made in duplicates in 5–7 samples in each group, and the data are represented as mean ± SD. *p < 0.05 vs. nondiabetic donors

Homocysteine conversion to SAM serves as a methyl donor for DNA methylation, and DNA methyl transferases (Dnmts) are redox-sensitive enzymes [11, 34]. The role of DNA methylation in the regulation of *CBS* and *MTHFR* gene transcripts in diabetic retinopathy was determined. Compared to nondiabetic donors, DNA at the promoters of both *CBS* and *MTHFR* was hypermethylated and DNMT1 was activated in the retinal microvessels from donors with diabetic retinopathy as observed by the 2-fold increase in 5mC levels at *CBS* promoter and ~ 2.5-fold increase at *MTHFR* promoter, and ~ 60% increase in *DNMT1* gene transcripts (Fig. 5a-c).

**Fig. 6:**
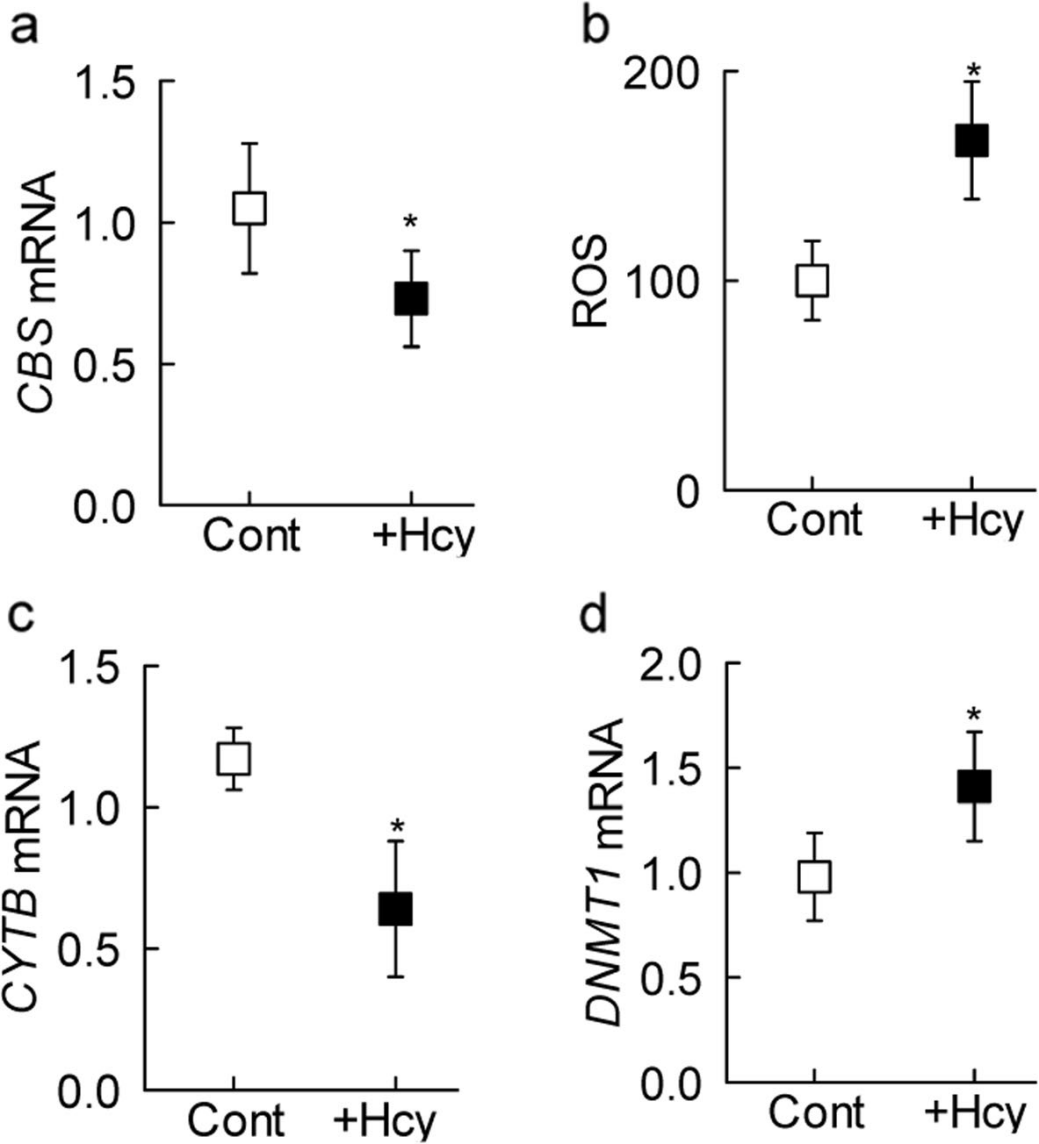
Effect of homocysteine supplementation on oxidative stress and DNA methylation machinery in isolated human retinal endothelial cells. HRECs, incubated in a medium containing homocysteine were analyzed for (**a**) *CBS* gene transcripts by q-RTPCR, (**b**) ROS levels by DCFH-DA method, and (**c**) *CYTB* and (**d**) *DNMT1* gene transcripts by q-RTPCR. β-actin was employed as a housekeeping gene for all q-RTPCR measurements. Results are represented as mean ± SD from 3 to 4 different cell preparations, with each measurement made in duplicates. Cont and + Hcy = cells incubated in normal incubation medium and normal incubation medium containing homocysteine, respectively. *p < 0.05 vs Cont

To confirm the specific effect of homocysteine, key parameters were analyzed in the HRECs incubated in the presence of homocysteine. As shown in Fig. 6a, homocysteine decreased *CBS* mRNA, and this was accompanied by increased oxidative stress and mitochondrial damage; ROS levels were ~ 70% higher and the gene transcripts of mtDNA-encoded *CYTB* were 40% lower in HRECs incubated in the presence of homocysteine, compared to without homocysteine (Fig. 6b and c). Similarly, the expression of *DNMT1* was also increased by homocysteine (Fig. 6d).

## Discussion

Retinopathy remains one of the major complications, which a diabetic patient fears the most. The pathogenesis of this blinding disease is very complex involving many inter-related molecular, biochemical, functional and structural alterations [1, 3, 4]. Although hyperglycemia is considered as the main instigator of its development, systemic factors including hyperlipidemia and blood pressure are also intimately associated with the development of diabetic retinopathy [35]. Nondiabetic normal population generally has > 15 μM plasma homocysteine, but in diabetic patients, they can go as high as 50-100 μM [10, 11]. High homocysteine in diabetic patients is associated with increased macular thickness without macular edema [36], and in patients with retinopathy, high homocysteine is considered to act as a common link through which other systemic factors could exert their deleterious effect on the progression of diabetic retinopathy [6, 37]. Homocysteinemia also results in photoreceptor degeneration [38], which is commonly seen in diabetes [39]. Here, our exciting data show that compared to nondiabetic controls, retina from human donors with established diabetic retinopathy have more than 3-fold higher homocysteine levels and significantly lower H_2_S, and a compromised machinery to transulfurnate and remethylate homocysteine. Diabetic donors also have impaired mitochondrial homeostasis with decreased transcription of mtDNA, imbalanced fusion-fission machinery and increased mitophagy markers. Their DNA methylation machinery is upregulated, and DNA hypermethylation of *CBS* and *MTHFR* promoters appears to be responsible for a compromised transsulfuration and remethylation machinery. These results clearly imply the importance of homocysteine in the development of diabetic retinopathy.

Homocysteine is a sulfur-containing amino acid, and its high circulating levels are considered as a risk factor for many diseases including heart disease and diabetic complications [7, 9]. Moderate increase in circulating homocysteine is considered to play a role in retinal abnormalities including endothelial cell dysfunction, ischemia, thinning of nerve fiber layers, neovascularization and blood-retinal barrier breakdown, the abnormalities intimately associated with diabetic retinopathy [40, 41]. Our results show that the donors with established diabetic retinopathy have higher homocysteine levels in their retinal microvasculature, the site of retinal histopathology characteristic of diabetic retinopathy.

Homocysteine removal, as mentioned above, is normally facilitated by two key processes, transsulfuration process converting homocysteine to cystathionine and eventually to cysteine, and homocysteine for synthesizing methionine in the methyl cycle [16, 17]. Inhibition of CBS and MTHFR, along with deficiencies in folate and vitamin B12, are considered as the primary causes of hyperhomocysteinemia [42]. Results presented here clearly demonstrate that donors with diabetic retinopathy have decreased levels of both CBS and MTHFR. Furthermore, retinal microvessels from donors with diabetic retinopathy also have decreased transcription of *CSE*, an enzyme responsible for breaking down cystathionine into cysteine, suggesting that the retinal microvasculature have the entire transsulfuration machinery and remethylation process impaired in diabetic retinopathy. In support, others have observed decreased CSE expression in endothelial cells and vascular smooth muscle cells in diabetic mice [43].

Transsulfuration of homocysteine is also closely associated with H_2_S production, and dysregulated transsulfuration machinery decreases H_2_S levels [16, 44]. Although H_2_S, a pungent smelling gas, has many toxic effects, it is now also considered an important signaling molecule (third gaseous) with important roles in a wide range of physiological and pathological conditions [45, 46]. Imbalance between homocysteine and H_2_S increases oxidative stress, nitric oxide levels, inflammation and ischemia/reperfusion injury [47]. Here, our results show that donors with diabetic retinopathy have decreased retinal H_2_S production and GSH levels. In support, homocysteine-H_2_S imbalance was shown to decrease cysteine, an amino acid critical for GSH biosynthesis [32]. Furthermore, we show here that incubation of isolated retinal endothelial cells with homocysteine increases oxidative stress and increased oxidative stress damages the retinal mitochondria and its DNA, as seen by decreased levels of mtDNA-encoded *CYTB*.

Mitochondrial homeostasis plays an important role in the pathogenesis of diabetic retinopathy, and experimental models have shown impaired mitochondrial dynamics [2–5]. Homocysteine plays a crucial role in reducing mitochondrial respiration and damaging the mitochondrial fusion-fission process [48]. CBS^+/−^ mice compared with wild-type mice, have increased mitochondrial fission, and their mitochondria are smaller in size [12]. Our present data show that retinal microvasculature from donors with diabetic retinopathy have an imbalance in the mitochondrial fusion-fission; they have high levels of mitochondrial fission protein Drp1, and suboptimal levels of the inner membrane fusion protein Mfn2. Furthermore, the mitophagy markers LC3 and OPTN are also higher in the retinal microvasculature from donors with diabetic retinopathy.

Homocysteine is also associated with global DNA methylation and *CBS*^*+/−*^ mice have increased Dnmts [34]. Increased DNA methylation is considered to suppress gene expression [49, 50], and experimental models have clearly shown activation of DNA methylation machinery in the retinal vasculature in diabetes [30]. Higher Dnmt1 and hypermethylation of the promoters of both *CBS* and *MTHFR* in the retinal microvessels from donors with diabetic retinopathy suggest that decreased *CBS* and *MTHFR*, seen in diabetic retinopathy donors, could be due to increased methylated cytosine levels at their promoters, impeding the binding of the transcription factors, and suppressing their gene expressions.

Homocysteine levels are also influenced by lifestyle including smoking and alcohol consumption [51, 52]. Although we do not accept eye globes from donors with any malignancy and drug use within the past 5 years, the possibility of other lifestyle factors influencing homocysteine levels in the donors used in present study cannot be ruled out. Diabetic retinopathy is a progressive disease, and although our inclusion criteria for diabetic donors requires the presence of retinopathy, this does not allow us to compare the homocysteine levels, and its metabolism, at different stages of diabetic retinopathy. Despite some limitations, our study provides convincing data documenting the importance of homocysteine in the development of diabetic retinopathy.

## Conclusions

Homocysteine is a common amino acid but its high levels are associated with many metabolic abnormalities and pathological conditions. This is the first report demonstrating that the machinery responsible for maintaining homocysteine levels in the retina is impaired in human donors with established diabetic retinopathy, increasing homocysteine levels in the retina and its microvasculature. The enzymes critical in transsulfuration and in remethylation are suboptimal, and the conversion of homocysteine to both cystathionine and methionine is impaired; the retina experiences a double whammy. H_2_S and GSH levels are decreased, and retinal mitochondria are damaged. Mechanistic insight into the suboptimal functioning of these enzymes suggests a critical role of epigenetic modifications; the promoters of both *CBS* and *MTHFR* have hypermethylated DNA. Interestingly, homocysteine itself also plays a major role in DNA methylation, and hypermethylation of *CBS* and *MTHFR* further interferes with the proper removal of homocysteine.

Thus, regulation of homocysteine levels in diabetic patients should prevent increase in retinal damage, and by regulating DNA methylation status of the enzymes responsible for the removal of homocysteine, should ameliorate further accumulation of this damaging sulfur containing non-protein amino acid. Impaired homocysteine metabolism is considered as the major cause of hyperhomocysteinemia. Folic acid and vitamin B12 are closely associated with maintaining homocysteine metabolism, and their supplementation reduces hyperhomocysteinemia [53]. This opens the possibility of using folic acid/vitamin B12 to potentially prevent/retard retinopathy in diabetic patients and alleviate their risk of losing vision.

## Data Availability

Not applicable.

## Abbreviations

5mC: 5-Methylcytosine
CBS: Cystathionine β-synthase
CSE: Cystathionine γ-lyase
CYTB: Cytochrome B
Dnmts: DNA methyltransferases
DRP1: Dynamin related protein 1
GSH: Glutathione
H_2_S: Hydrogen sulfide
HRECs: Human Retinal Microvascular Endothelial Cells
LC3: Microtubule-associated protein 1A/1B-light chain 3
MFN2: Mitofusin-2
mtDNA: Mitochondrial DNA
MTHFR: Methylenetetrahydrofolate reductase
NaHS: Sodium hydrosulfide
OPTN: Optineurin
q-RTPCR: Quantitative real-time PCR
SAM: S-adenosylmethionine
