# Correction to: Faulty homocysteine recycling in diabetic retinopathy

**DOI:** 10.1186/s40662-020-00178-3

**Published:** 2020-02-28

**Authors:** Renu A. Kowluru, Ghulam Mohammad, Nikhil Sahajpal

**Affiliations:** Department of Ophthalmology, Visual Sciences and Anatomical Sciences, Wayne State University, 4717 St. Antoine, Detroit, MI 48201 USA

**Correction to: Eye and Vision (2020) 7:4**

**https://doi.org/10.1186/s40662-019-0167-9**

In the original publication of this article [1] Fig. 2b is incorrect. The correct Fig. 2 is as below. The original publication has been corrected.

**Fig. 2:**
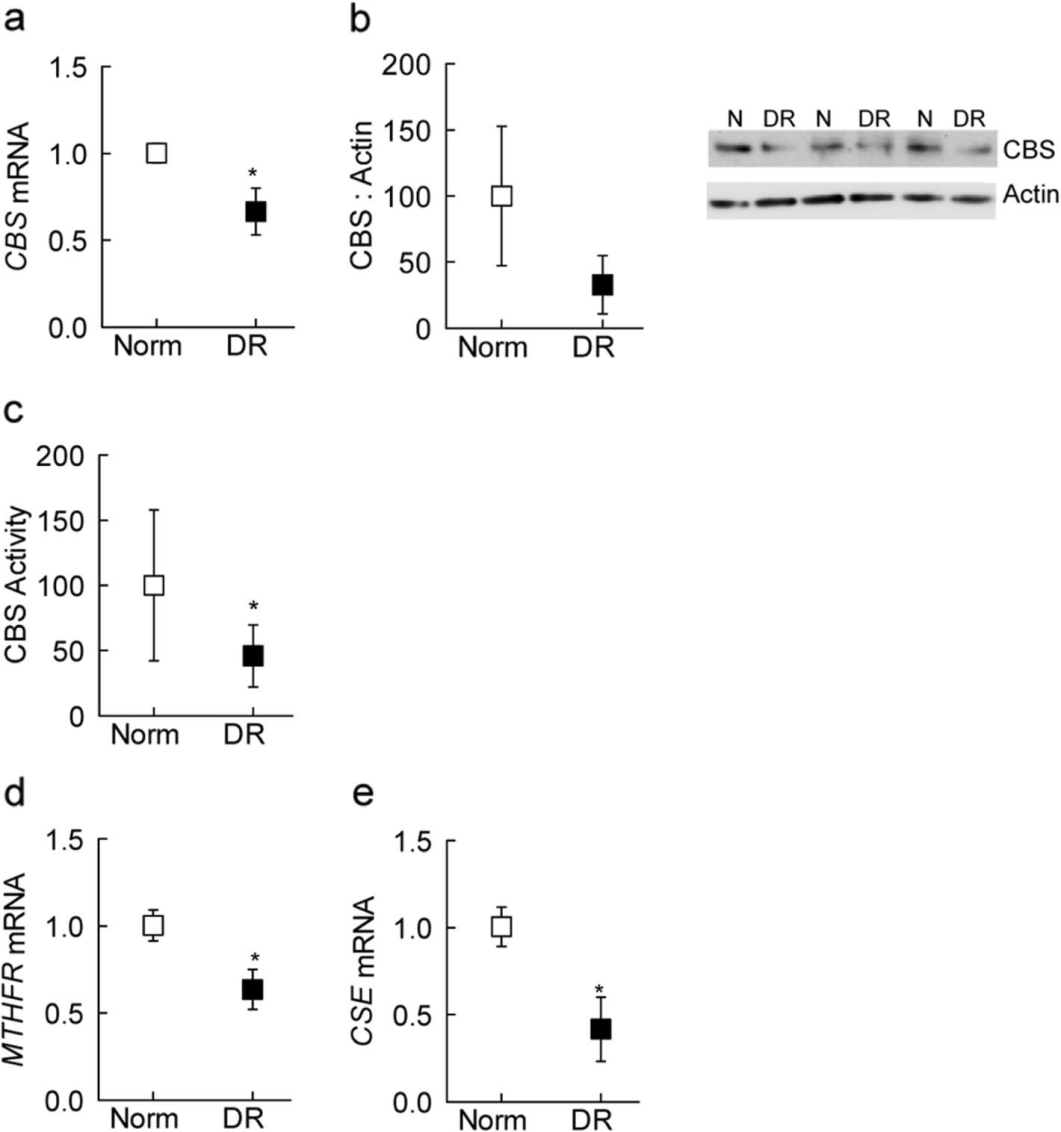
Homocysteine metabolizing machinery in diabetic retinopathy. Retinal microvessels were employed to determine CBS (**a**) gene transcripts by q-RTPCR, (**b**) protein expression by Western blotting, using β-actin as a housekeeping gene and loading protein, respectively, and (**c**) enzyme activity by measuring fluorescence at 368 nm excitation and 460 nm emission wavelengths. Values obtained from nondiabetic controls are considered as 100%. Gene transcripts of (**d**) *MTHFR* and (**e**) *CSE* were quantified by q-RTPCR using β-actin as a housekeeping gene. Data are represented as mean ± SD, obtained from retinal microvessels from 6 to 8 nondiabetic and 7–8 diabetic retinopathy donors. *p < 0.05 vs. nondiabetic donors
